# *Lactobacillus acidophilus* Alleviates Platelet-Activating Factor-Induced Inflammatory Responses in Human Intestinal Epithelial Cells

**DOI:** 10.1371/journal.pone.0075664

**Published:** 2013-10-09

**Authors:** Alip Borthakur, Sumit Bhattacharyya, Anoop Kumar, Arivarasu Natarajan Anbazhagan, Joanne K. Tobacman, Pradeep K. Dudeja

**Affiliations:** 1 Division of Gastroenterology and Hepatology, Department of Medicine, University of Illinois at Chicago, Chicago, Illinois, United States of America; 2 Jesse Brown VA Medical Center, Chicago, Illinois, United States of America; UCSF/VA Medical Center, United States of America

## Abstract

Probiotics have been used as alternative prevention and therapy modalities in intestinal inflammatory disorders including inflammatory bowel diseases (IBD) and necrotizing enterocolitis (NEC). Pathophysiology of IBD and NEC includes the production of diverse lipid mediators, including platelet-activating factor (PAF) that mediate inflammatory responses in the disease. PAF is known to activate NF-κB, however, the mechanisms of PAF-induced inflammation are not fully defined. We have recently described a novel PAF-triggered pathway of NF-κB activation and IL-8 production in intestinal epithelial cells (IECs), requiring the pivotal role of the adaptor protein Bcl10 and its interactions with CARMA3 and MALT1. The current studies examined the potential role of the probiotic *Lactobacillus acidophilus* in reversing the PAF-induced, Bcl10-dependent NF-κB activation and IL-8 production in IECs. PAF treatment (5 µM×24 h) of NCM460 and Caco-2 cells significantly increased nuclear p65 NF-κB levels and IL-8 secretion (2-3-fold, *P*<0.05), compared to control, which were blocked by pretreatment of the cells for 6 h with *L. acidophilus* (LA) or its culture supernatant (CS), followed by continued treatments with PAF for 24 h. LA-CS also attenuated PAF-induced increase in Bcl10 mRNA and protein levels and Bcl10 promoter activity. LA-CS did not alter PAF-induced interaction of Bcl10 with CARMA3, but attenuated Bcl10 interaction with MALT1 and also PAF-induced ubiquitination of IKKγ. Efficacy of bacteria-free CS of LA in counteracting PAF-induced inflammatory cascade suggests that soluble factor(s) in the CS of LA mediate these effects. These results define a novel mechanism by which probiotics counteract PAF-induced inflammation in IECs.

## Introduction

Recent clinical and experimental outcomes have shown that intestinal luminal microbiota play pivotal role in the pathogenesis of inflammatory bowel diseases (IBD) and necrotizing enterocolitis (NEC) [1]–[4]. Therefore, modifying gut flora composition using probiotics has been emerging as a promising strategy to alleviate mucosal inflammation in these diseases [3], [5], [6]. Probiotic bacteria including *Lactobacilli* have been shown to interact with cells of the mucosal surface and locally modulate the production and/or activity of inflammatory mediators [7]. In this regard, platelet activating factor (PAF), a potent bioactive phospholipid known to cause intestinal injury [8], has been implicated in the pathogenesis of IBD [9], [10] and NEC [11], [12]. Elevated PAF levels have been reported in tissues and/or serum in response to pathogen infection, and in patients with Crohn’s disease, ulcerative colitis, and NEC, that correlated with disease severity [9], [10], [12], [13]. PAF acts by binding to and activating G-protein coupled PAF receptors (PAF-R). Highest concentrations of PAF-R expression have been reported in intestinal epithelium [14]. PAF is known to activate NF-κB, a key transcriptional regulator of the expression of proinflammatory cytokines and many immunoregulatory molecules in response to inflammatory stimuli [15]–[17] and microbial infection [18]. However, the early receptor-mediated signaling events that initiate these responses are not completely defined.

NF-κB activity is tightly regulated by interaction with inhibitory proteins, IκBs, which sequester NF-κB in cytoplasm. Upon stimulation, IκB is phosphorylated by the NF-κB-activating IκB kinase (IKK) complex, ubiquitinated, and degraded by the 26S proteasome complex, thereby releasing NF-κB to translocate into the nucleus and initiate specific target gene transcription [19]. Studies during the past two decades have revealed tissue-specific and stimulus-specific roles of many scaffold proteins in linking different receptors to the IKK complex [20], [21]. Among those, a family of caspase recruitment domain (CARD)-containing scaffold proteins, known as CARD- and membrane-associated guanylate kinase-like domain-containing protein (CARMA) plays critical roles in recruitment and activation of IKK. Bcl10 and MALT1 physically interact to activate NF-κB whereas CARMA proteins function as upstream regulators of Bcl10 and MALT1 in response to various stimuli in different tissues. Together, these three proteins constitute the CARMA/Bcl10/MALT1 (CBM) signalosome, which plays a critical role in regulating NF-κB activation both in normal physiology as well as in various pathophysiological conditions [22]. Originally described in immune cells [23], this pathway has been shown to mediate inflammatory responses in myeloid and epithelial cell types [22], including intestinal epithelial cells [24]–[26]. Importantly, we have recently reported a novel inflammatory pathway induced by PAF to activate NF-κB and produce IL-8 in human intestinal epithelial cells. This NF-κB-activating cascade involved upregulation of Bcl10 expression and its increased interactions with CARMA3 and MALT1 [24].

Multiple studies have shown the role of probiotic bacteria in ameliorating intestinal inflammation via inhibition of NF-κB activity [27], [28]. However, despite known involvement of PAF as an inflammatory mediator in IBD and NEC, there are no studies testing the efficacy of probiotics or probiotic-derived molecules in counteracting pro-inflammatory effects of PAF in diseases. Since PAF-induced NF-κB activation via CARMA3-Bcl10-MALT1 signalosome could highlight important targets for intervention, we investigated the effects of probiotic *Lactobacilli* in counteracting PAF-induced inflammation and explored the underlying mechanisms.

## Results

### *L. acidophilus* blocks PAF-induced NF-κB activation and IL-8 production in human intestinal epithelial cells

**Figure 1:**
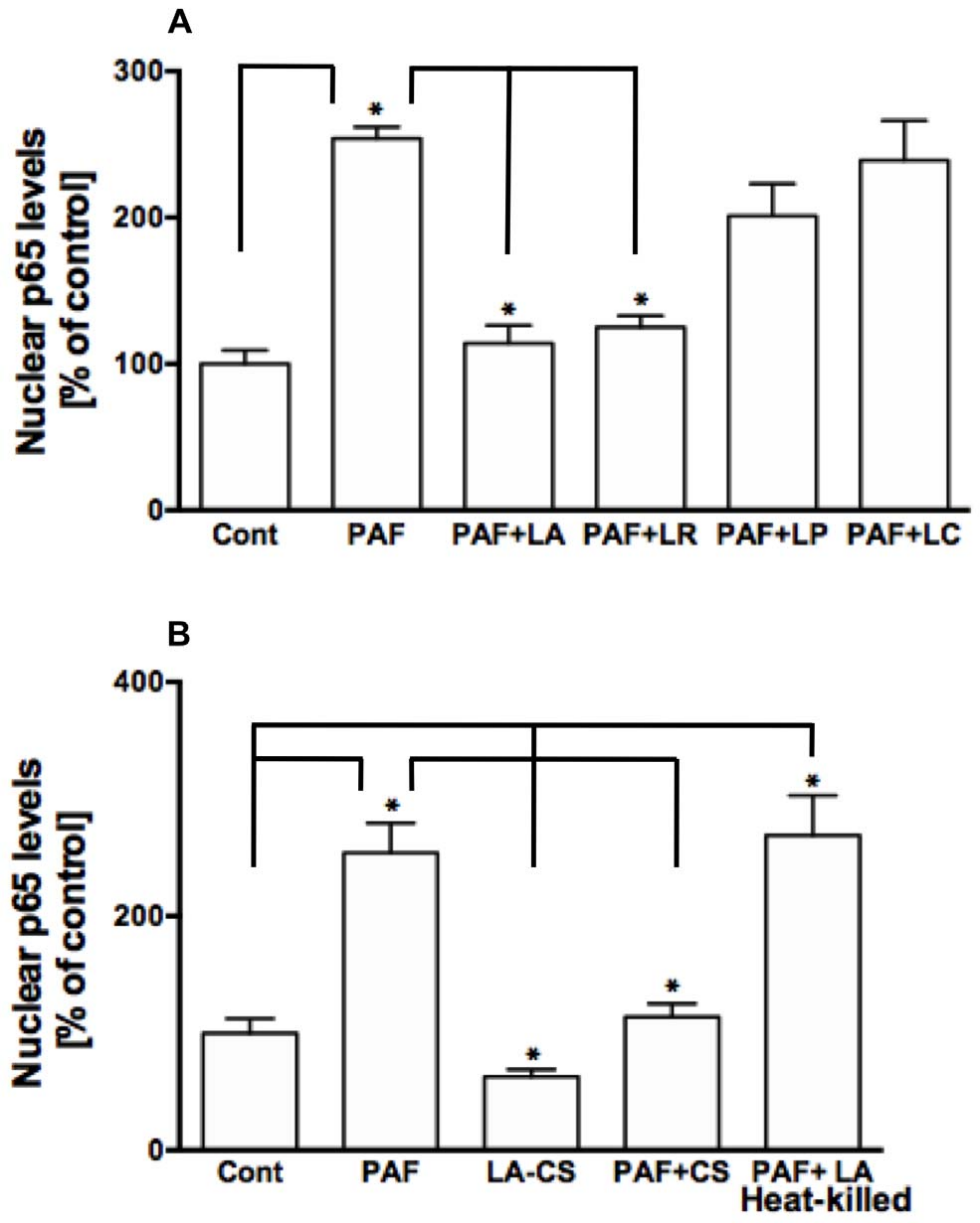
*L. acidophilus* and its culture supernatant counteract PAF-induced NF-κB activation in IECs. (**A**) Nuclear extracts of control or PAF ± *L. acidophilus* (LA), *L. rhamnosus* (LR), *L. plantarum* (LP) or *L. casei* (LC)-treated NCM460 cells for 24 h were utilized to measure p65 levels by oligonucleotide-based ELISA (n = 5, *P<0.05); (**B**) p65 levels in the nuclear extracts from control or PAF ± *L. acidophilus* culture supernatant (CS) or heat-killed LA-treated NCM460 cells (n = 3, *P<0.05).

**Figure 2:**
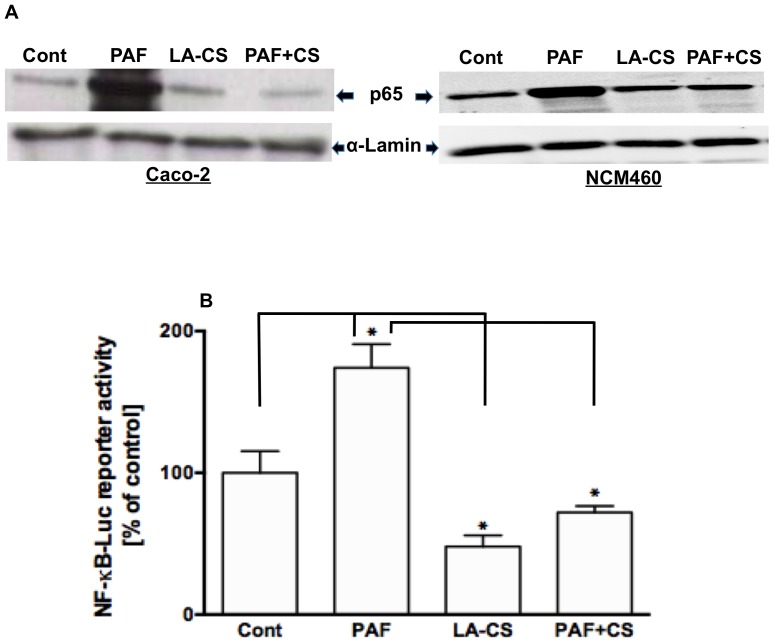
*L. acidophilus* culture supernatant attenuates PAF-induced increase in nuclear p65 and NF-κB reporter activity. (**A**) p65 levels measured by immunoblotting of nuclear extracts prepared from control or PAF ± LA-CS-treated Caco-2 and NCM460 cells (representative blots from n = 3); (**B**) NF-κB reporter activity, expressed as percent of control, in control or PAF ± LA-CS-treated Caco-2 cells (n = 4, *P<0.05).

We have previously shown that PAF has a specific direct effect on NF-κB activation and IL-8 secretion in human colonic NCM460 and Caco-2 cells [24]. In this study, we examined the efficacy of few important *Lactobacillus* species in blocking the effects of PAF on NF-κB activation. During NF-κB activation, inhibitory IκB proteins are phosphorylated by the IKK signalosome, thereby releasing NF-κB for nuclear translocation to activate target genes [19], [20]. Therefore, we used oligonucleotide-based ELISA and immunoblotting to measure NF-κB (p65) in the nuclear fractions isolated from control and treated NCM460/Caco-2 cells. NCM460 cells were pre-incubated for 6 h with one of the following *Lactobacillus* species: *L. acidophilus, L. rhamnosus, L. plantarum* and *L. casei* followed by 24 h further incubation with or without 5 µM PAF. NF-κB activation was assessed by measuring p65 levels in the nuclear extracts from NCM460 cells, control and different treatment groups, utilizing oligonucleotide probe-based ELISA. As shown in **Figure 1A**, the two species, *L. acidophilus* (LA) and *L. rhamnosus* (LR) significantly attenuated the PAF-induced increase in nuclear p65, whereas the other two species were ineffective. We, therefore, utilized only one species, LA, for all subsequent studies. We next examined the effects of heat-killed LA or its conditioned culture supernatant (CS) on nuclear p65 levels. Heat-killed LA failed to attenuate PAF-induced increase in nuclear p65, whereas, similar to live LA, its CS (diluted 1∶10 in DMEM/F12) was equally effective in attenuating the PAF effects on nuclear p65 (**Figure 1B**). A dose-dilution response of CS was initially performed and 1∶10 dilution was chosen to obtain the optimal effect and at the same time to avoid adverse effects of long-term incubation on cell viability as observed with lesser dilutions (1∶2 or 1∶5). These results indicate that LA effects on PAF-induced NF-κB activation are mediated by secreted soluble factor(s) in the CS. Therefore, for all subsequent experiments we used LA-CS instead of live LA for treatment of cells. Nuclear p65 levels as assessed by immunoblotting are shown in **Figure 2A**. PAF increased nuclear p65 in both NCM460 and Caco-2 cells, whereas LA-CS blocked the PAF effects on nuclear p65. We also measured NF-κB activity by transfecting Caco-2 cells with the NF-κB transcription reporter vector p-NF-κB-Luc (BD Biosciences). This vector contains NF-κB consensus sequence located upstream of the firefly luciferase reporter gene. After 16 h of stimulation, PAF (5 µM) activated NF-κB-dependent reporter gene in Caco-2 cells (∼2-fold compared to control). This increase was significantly attenuated in cells preincubated with LA-CS for 6 h, followed by continued treatments along with PAF. (**Figure 2B**).

### *L. acidophilus* attenuates PAF-induced I-κB phosphorylation

**Figure 3:**
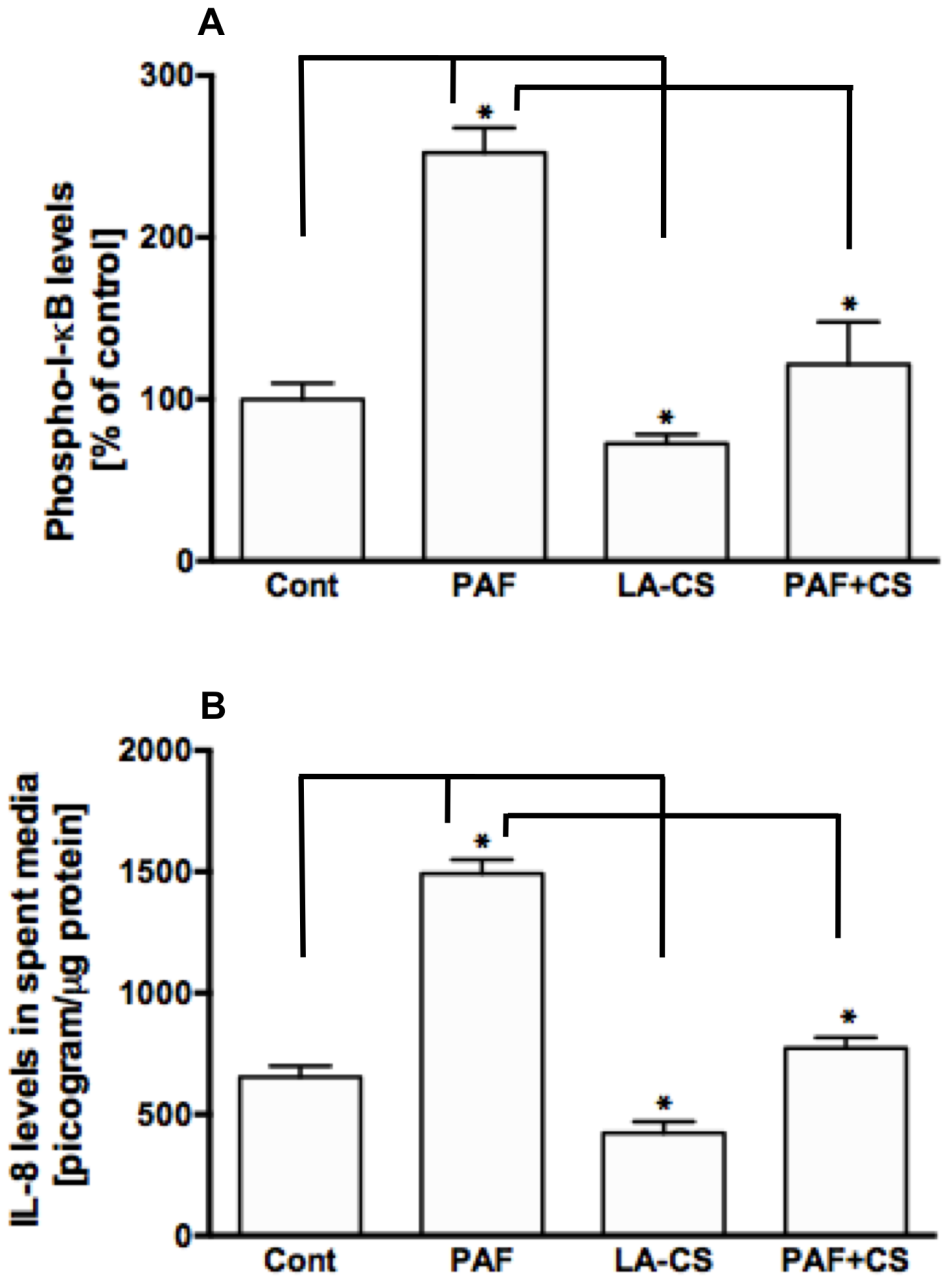
*L. acidophilus* culture supernatant attenuates PAF-induced I*-*κB phosphorylation and IL-8 production. (**A**) Phospho-I- κB levels, as measured by ELISA, in total lysates prepared from control or PAF ± LA-CS-treated NCM460 cells (n = 3, *P<0.05); (**B**) IL-8 level as measured by ELISA, in the spent media of control or PAF ± LA-CS-treated NCM460 cells (n = 3, *P<0.05).

We also measured phospho-IκB utilizing an ELISA-based method to assess the levels in response to PAF and/or LA-CS treatments. LA-CS significantly attenuated phospho-IκB levels compared to control and also blocked the PAF-induced increase in phospho-IκB levels in NCM460 cells (**Figure 3A**).

### *L. acidophilus* blocks PAF-induced IL-8 production

We next tested whether the PAF-induced increase in IL-8 in the spent media, as reported earlier [24], was reversed by LA-CS treatments. LA-CS alone did not affect IL-8 levels in the spent media of NCM460 cells, as measured by ELISA. However, pre- and continued incubation of the cells with LA-CS significantly attenuated the PAF-induced IL-8 in the spent media (**Figure 3B**)

### LA-CS attenuated PAF-induced increase in Bcl10 mRNA and protein expression, and Bcl10 promoter activity

**Figure 4:**
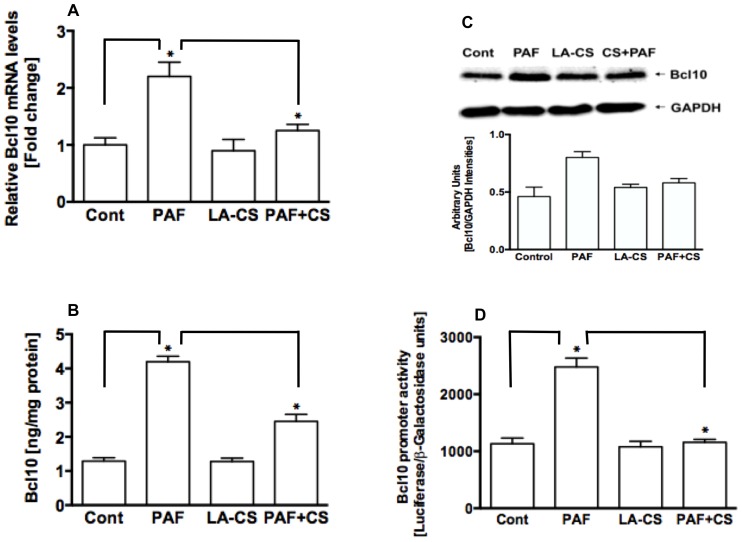
*L. acidophilus* culture supernatant does not alter Bcl10 expression but attenuates PAF-induced increase in Bcl10 expression in IECs. (**A**) relative levels of Bcl10 mRNA in control or PAF ± LA-CS-treated NCM460 cells measured by real-time RT-PCR as described in Methods (n = 3, *P<0.05); (**B**) Bcl10 protein levels measured by ELISA in control or PAF ± LA-CS-treated NCM460 cells (n = 3, *P<0.05); (**C**) representative blot (n = 3) of Bcl10 protein levels measured by immunoblotting (upper panel) in control or PAF ± LA-CS-treated Caco-2 cells and densitometric analysis of the relative band intensities (lower panel); (**D**) Bcl10 promoter activity in control or PAF ± LA-CS-treated Caco-2 cells (n = 4, *P<0.05).

Our previous studies showed that PAF treatments caused a dose-dependent increase in Bcl10 protein expression, and that Bcl10 was required for PAF-induced NF-κB activation and IL-8 secretion [24]. Therefore, we sought to investigate whether LA-CS, which blocked PAF-induced NF-κB activation and IL-8 production, also regulates expression of Bcl10. LA-CS alone had no effects on Bcl10 mRNA levels (**Figure 4A**), Bcl10 protein, as measured by ELISA (**Figure 4B**) and immunoblotting (**Figure 4C**) in NCM460 cells, and Bcl10 promoter activity (**Figure 4D**) in Caco-2 cells. However, pre-treatment of the cells with LA-CS significantly attenuated the PAF-induced increase in Bcl10 mRNA and protein expression and Bcl10 promoter activity.

### Probiotic supernatants reduced PAF-induced Bcl10-MALT1 interaction and ubiquitination of IKKγ

**Figure 5:**
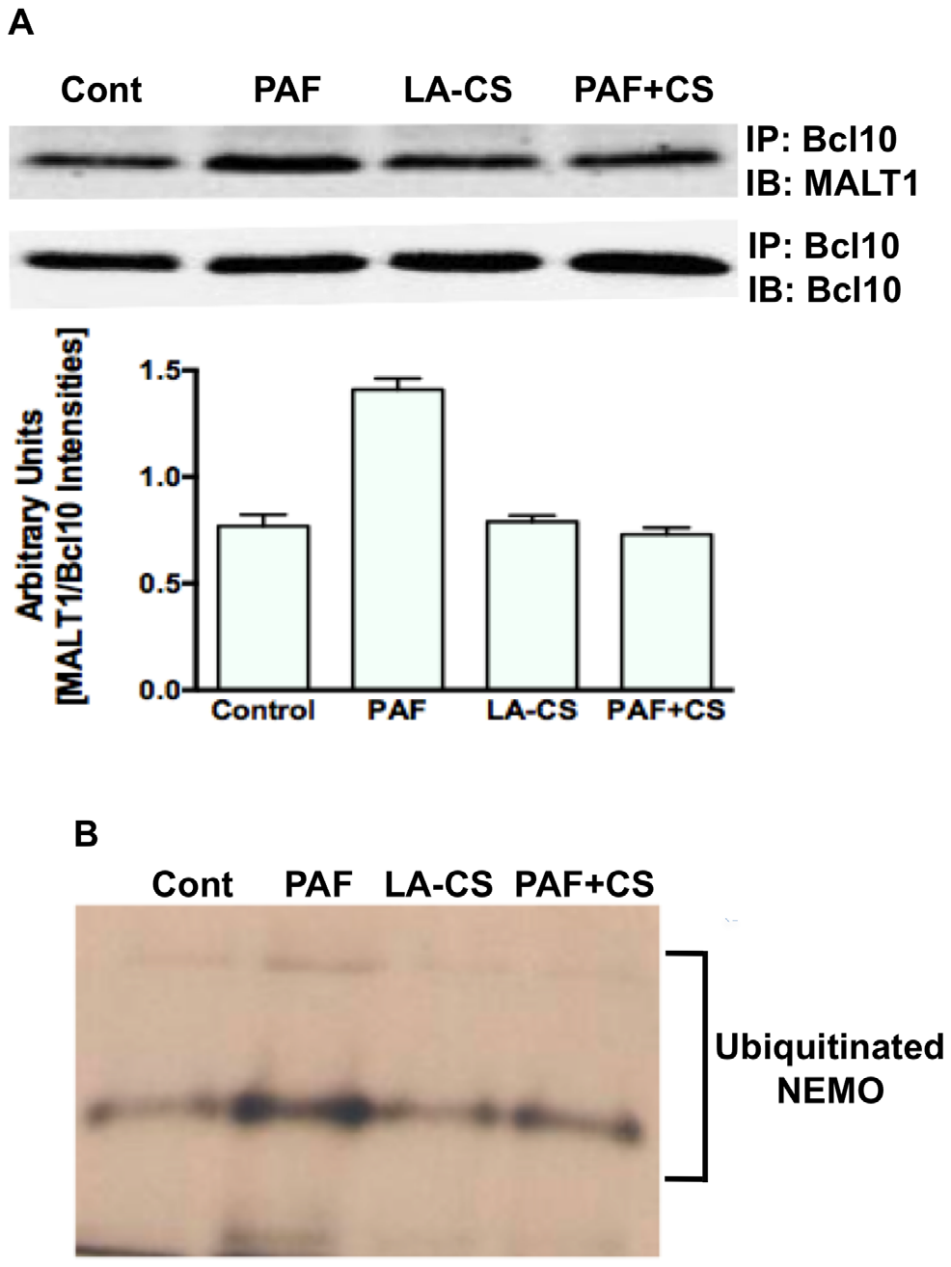
*L. acidophilus* culture supernatant attenuates PAF-induced Bcl10 interaction with MALT1 and ubiquitination of IKKγ (NEMO). (**A**) Cell lysates of control or PAF ± LA-CS-treated NCM460 cells, containing equal amounts of proteins, were used to immunoprecipitate (IP) MALT1 with anti-Bcl10 antibody. Immunoprecipitates were subjected to SDS-PAGE and probed with anti-MALT1 antibody in immunoblotting (IB). After stripping with 0.2N NaOH, blots were re-probed with anti-Bcl10 antibody; upper panel: representative blot of 3 independent experiments; lower panel: densitometric analysis of relative band intensities; (**B**) Caco-2 cells co-transfected with expression vectors encoding pcDNA3-IKKγ-Myc and pcDNA3-ubiquitin-HA were untreated or treated with PAF ± LA-CS as described in Methods. After purification by anti-Myc antibody immunoprecipitation, the IKKγ protein was assayed for ubiquitination by Western blotting with anti-HA antibody.

Recent reports from our laboratory and others [22], [24]–[26] showed that Bcl10-dependent inflammatory pathways in nonimmune cells involve the CARMA-3/Bcl10/MALT1 signalosome complex. Our previous studies also showed that PAF increased interaction of Bcl10 with CARMA3 and MALT1 [24]. Therefore, we used co-immunoprecipitation studies to examine the effects of preincubating NCM460 cells with LA-CS on the PAF-induced interactions of Bcl10-CARMA3-MALT1. LA-CS did not alter PAF-mediated enhancement of Bcl10-CARMA3 interactions (not shown), but significantly reduced PAF-induced interaction of Bcl10 and MALT1 (**Figure 5A**). Specificity of Bcl10/MALT1 co-immunoprecipitation was confirmed by incubation of the cell lysate with isotype specific rabbit IgG instead of anti-Bcl10 antibody that failed to immunoprecipitate MALT1 (not shown).

We next sought to analyze the effects of PAF on ubiquitination of IKKγ, the regulatory subunit of IKK complex, which is reported to be important in Bcl10-mediated NF-κB activation [29]. Caco-2 cells were transfected with recombinant constructs, Myc-tagged IKKγ and HA-tagged ubiquitin. Ubiquitinated IKKγ was immunoprecipitated with anti-Myc antibody and probed with anti-HA antibody in immunoblots to assess ubiquitination. PAF treatment increased ubiquitination of IKKγ (∼2-fold), which was attenuated in cells preincubated with LA-CS prior to PAF treatments (**Figure 5B**)

## Discussion

Gut bacteria play a key role in inflammatory bowel diseases (IBD) and necrotizing enterocolitis (NEC) [1]–[4]. Although much attention has been focused on the search for a pathogen or inciting inflammatory bacteria, of equal importance is the search for beneficial commensal bacteria that normally confer anti-inflammatory effects in the gut. Probiotics, a group of gut commensals that confer health benefits are known to ameliorate inflammation and has been used in clinical trials and experimental models to alleviate inflammation in IBD and NEC [30]–[33]. However, the nature of the probiotic-derived active components that mediate their beneficial effects and underlying mechanisms of action has not been fully elucidated. Pathophysiology of IBD and NEC involves production of diverse lipid mediators, platelet-activating factor (PAF) being one of them mediating inflammatory responses in these diseases [9]–[11], [30]. PAF is produced by the human intestinal epithelium [14], where it mediates a range of biological effects such as modulation of ion transport, prostaglandin and eicosanoid synthesis and apoptosis [34]–[39]. On the other hand, PAF has also been shown to trigger inflammatory responses via NF-κB activation [12], [15], [38] and cytokine and chemokine gene expression in a wide variety of cells [17], [40], [41]. However, the signaling events that mediate PAF-induced NF-κB activation are not completely defined. In this regard, we have recently described a novel inflammatory pathway induced by PAF to activate NF-κB and produce IL-8 in NCM460, a cell line derived from normal human colon, and Caco-2, a transformed human intestinal cell line [24]. Our studies showed that direct *in vitro* activation of NF-κB by PAF in intestinal epithelial cells required Bcl10, an adaptor protein, and its interactions with CARMA3 and MALT1 [24]. The hallmark of antigen receptor-induced NF-κB activation in lymphocytes has been shown to be the formation CARMA1-Bcl10-MALT1 signalosome, which directly or indirectly activates I-κB kinase (IKK) complex to phosphorylate I-κB proteins thereby releasing NF-κB for nuclear translocation [23]. In non-immune cells, however, this pathway utilizes CARMA3, a second member of the CARMA family having a wider tissue distribution [42]. PAF-induced IκBα phosphorylation, NF-κB activation, and IL-8 production in NCM460 and Caco-2 cells were Bcl10-dependent. PAF upregulated Bcl10 expression in these cells via transcriptional mechanisms and enhanced its interactions with CARMA3 and MALT1 [24]. Since proinflammatory effects of PAF play prominent roles in the pathogenesis of IBD and NEC, it was of great interest to investigate the effects of probiotic *Lactobacilli* in counteracting PAF-induced NF-κB activation via CARMA3-Bcl10-MALT1 signalosome and to elucidate the underlying mechanisms. Our results demonstrated that *Lactobacillus acidophilus*, an important gut commensal, counteracted PAF induction of Bcl10-dependent NF-κB activation and IL-8 production in intestinal epithelial NCM460 and Caco-2 cells. Earlier we have shown that this specific strain of *L. acidophilus,* and more importantly its conditioned culture supernatant, exhibited pro-absorptive effects via distinct mechanisms to stimulate intestinal absorption of NaCl, thereby defining its novel therapeutic potential to ameliorate diarrhea associated with IBD [43], [44]. Interestingly, our current studies also showed that heat-killed LA failed to attenuate PAF-induced increase in nuclear p65 and importantly, bacteria-free CS of LA was as effective as live LA in attenuating the PAF effects on nuclear p65, indicating that LA effects on PAF-induced NF-κB activation are mediated by secreted soluble factor(s) in the CS. LA-CS also attenuated basal NF-κB reporter activity as well as completely blocked PAF-mediated increase in NF-κB reporter activity. Further, LA-CS not only attenuated IL-8 secretion and I-κB phosphorylation compared to control, but also blocked PAF-induced increase in IL-8 secretion and I-κB phosphorylation. Various earlier studies have shown the importance of bacteria-secreted soluble factors, cell wall components, and bacterial DNA as mediators of the beneficial effects of bacteria [45]–[48]. These studies are of great significance for eliminating the concerns in using live bacteria to treat diseases like IBD. However, determining the exact chemical nature of the bacteria-derived factors and elucidating their mechanisms of action will only help design targeted therapies for these inflammatory diseases.

**Figure 6:**
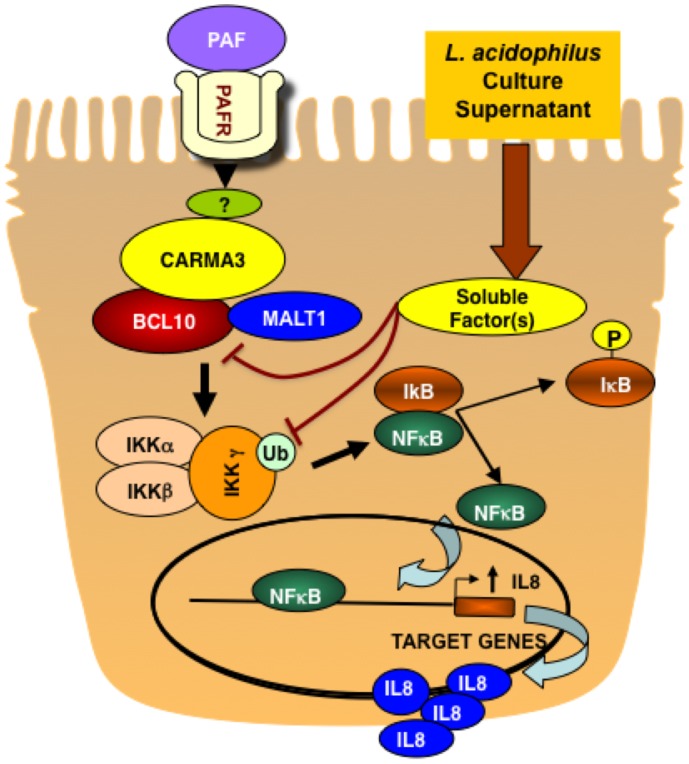
Model showing mechanisms implicated in alleviating PAF-induced NF-κB activation and IL-8 production in IECs by LA-derived soluble factors.

Our previous studies showed that PAF-induced increase in NF-κB activation and IL-8 production critically required Bcl10 and were associated with PAF-induced increase in Bcl10 gene transcription [24]. On the other hand, LA-CS did not alter Bcl10 mRNA and protein levels or Bcl10 promoter activity, but attenuated PAF-induced increase in Bcl10 mRNA, protein and promoter activity. Since LA-CS alone showed no effects on Bcl10 transcription, its effects in attenuating PAF-induced increase in I-κB phosphorylation, NF-κB activation, and IL-8 production could involve different mechanisms. LA-CS significantly blocked Bcl10 interactions with MALT1, but had no effect on its interactions with CARMA3, the scaffold protein known to act upstream of Bcl10 and MALT1. Previous studies have reported that in the T-cell receptor pathway involving CARMA1/Bcl10/MALT1, interaction with MALT1 (a paracaspase) brings Bcl10 closer to IKK complex, and that Bcl10 activates NF-κB pathway through ubiquitination of IKKγ (NEMO), the regulatory subunit of IKK complex [29]. NEMO ubiquitination has been suggested to represent a way to attract the IKK complex to upstream activators and critical for canonical NF-κB activation pathway [49]. Interestingly, our current studies showed increased NEMO ubiquitination in response to PAF treatments, whereas LA-CS substantially attenuated this effect of PAF. These results suggest that LA-CS could ameliorate PAF-induced intestinal inflammatory responses via modulation of signaling events associated with activation of IKK complex, rather than either the early events following receptor activation by PAF or later events downstream of IKK signalosome (**Figure 6**).

In essence, our current set of studies suggests a distinct mechanism of functionality of probiotic-derived molecules to combat PAF-induced intestinal inflammation at the epithelial cell level. Our future studies to precisely define the underlying mechanisms of LA-CS attenuation/reversal of PAF-induced inflammatory cascade will further increase our understanding of molecular basis of these effects in ameliorating intestinal inflammation. Further, molecular characterization of the LA-secreted soluble factors mediating these effects will be of great clinical significance for the management of IBD and/or NEC.

## Materials and Methods

### Materials, reagents and antibodies

PAF (1-O-alkyl-2-acetyl-sn-glycero-3-phosphocholine) was obtained from Sigma-Aldrich (St. Louis, MO). Antibodies specific for Bcl10 (Cat# sc-13153), CARMA3 (Cat# sc-47826), MALT1 (Cat# sc-46677) and NF-κB p65 (Cat# sc-8008) were purchased from Santa Cruz Biotechnology (Santa Cruz, CA).

### Cell lines, Cell culture and Treatments

The human colonic epithelial cell line NCM460, derived from normal colonic mucosa, was grown in M3:10 medium (INCELL, St. Antonio, TX) and maintained at 37°C in a humidified, 5% CO_2_ environment. Caco-2 cells were maintained in DMEM with 4.5 g/L glucose, 50 kU/L penicillin, 5 mg/L streptomycin, and 20% fetal bovine serum. For experiments, confluent cells in cell culture flasks were trypsinized and seeded into 24-well plates at a cell density of 2×10^4^ cells/ml. At 60–70% confluency, cells were used for treatments. Serum was reduced to 1% for overnight before treatments and also during the treatments.

### Bacterial culture and preparation of conditioned medium

The following probiotic *Lactobacillus* species, with strain numbers given in parentheses, were obtained from American Type Culture Collection (ATCC): *L. acidophilus* (4357), *L. rhamnosus* (53103), *L. plantarum* (14917) and *L. casei* (393). They were grown in Mann-Rogosa-Sharpe broth (Difco) for 24 h at 37°C without shaking. The overnight culture was centrifuged at 3000 x g for 10 min at 4°C. The supernatant, filtered through a 0.22-µm filter (Millex, Millipore) to sterilize and remove all bacterial cells, was designated as conditioned medium (CS). For treating the cells with live bacteria, the bacterial pellet was washed with DMEM/F-12 media (Invitrogen) containing 5 mg/L mannose and resuspended in the same media.

### Treatment of cells

NCM460 or Caco-2 cells grown on 24-well plates to 60–70% confluency were pre-treated for 6 h either with live bacteria suspended in DMEM/F-12 media (0.5×10^7^ CFU per well) or with CM diluted 1∶10 in the same media. The 1∶10 dilution was chosen to obtain the optimal effect and at the same time to avoid adverse effects of long-term incubation on cell viability as observed with lesser dilutions (1∶2 or 1∶5). For subsequent co-incubation with or without PAF for 24 h, the CFU of live bacteria used was reduced to 0.5×10^6^ per well whereas CS was used at the same dilution (1∶10). Changes of pH towards acidic range upon addition of culture supernatant to DMEM/F12 were normalized with 0.5N NaOH before incubation.

### RNA extraction and real-time RT-PCR

The total RNA from NCM460 cells was prepared using RNeasy Mini Kit (Qiagen, Valencia, CA) according to manufacturer’s instructions. An equal amount of RNA for each sample was reverse-transcribed and amplified in a one-step reaction using Brilliant SYBR Green QRT-PCR master mix kit (Stratagene, La Jolla, CA) and using Mx 3000 (Stratagene). The gene specific primers for human Bcl10 (5′-3′), were, forward: AAGGTCTGGACACCCTTGTT and reverse: ACAGTGGATGCCCTCAGTTT. The quantification of the amplification was expressed as a ratio of 2^ΔCt-Bcl10^/2^ΔCt-β-actin^, where ΔCt-Bcl10 and ΔCt-β-actin represent the difference between the threshold cycles of amplification of Bcl10 and β-actin.

### Immunoblotting

Proteins in the NCM460 or Caco-2 cell lysates were separated by SDS-PAGE on an 8% gel. Proteins were transferred to a nitrocellulose membrane (Amersham Biosciences, Piscataway, NJ) and probed with the Bcl10 antibody. Immunoreactive bands were visualized using the ECL detection kit (Amersham).

### Co-immunoprecipitation

Control NCM460 cells and cells treated with PAF, LA-CM with or without PAF were washed in cold PBS and lysed in a lysis buffer [20 mM Tris pH 7.5, 150 mM NaCl, 1% TritonX-100, 1 mM EDTA, 30 mM NaF, 2 mM sodium pyrophosphate and 1X protease inhibitor cocktail (Roche)]. The cell lysates were pre-cleared with protein A/G plus-agarose (Santa Cruz) and then incubated with anti-CARMA3 or anti-MALT1 antibodies at 4°C for 16 h followed by incubation with protein A/G plus-agarose for 5 h. The agarose beads were collected by centrifugation, washed 4 times with lysis buffer and heated to 95°C for 5 min after adding Laemmli buffer. The resulting immunoprecipitates were separated by SDS-PAGE and probed with anti-Bcl10 antibody in immunoblotting.

### Preparation of nuclear extract and measurement of p65

Nuclear extracts from control and treated NCM460 or Caco-2 cells were prepared using the nuclear extraction kit from Active Motif following the manufacturer’s protocol as described earlier by us [26]. NF-κB activation was assessed by measuring levels of nuclear p65 by oligonucleotide-based ELISA as previously described by us [26], or by immunoblotting with anti-p65 antibody.

### NF-κB activity

NF-κB activation in response to PAF with or without pre-incubation and co-incubation with LA-CM was measured by NF-κB-Luciferase reporter assay as described earlier [24]. Briefly, NCM460 or Caco-2 cells were transfected with p-NF-κB-Luc (Clontech, CA) using Lipofectamine 2000 reagent (Invitrogen, Carlsbad, CA). This plasmid contains NF-κB binding consensus element upstream of luciferase reporter gene. Twenty-four hours after transfection, cells were treated with PAF (10 µM) with or without pre- and co-treatments with LA-CM for another 24 h. Luciferase assays were performed and results were expressed as RLU/mg protein.

### ELISA for Bcl10

The levels of Bcl10 were determined by a solid-phase sandwich ELISA, as previously developed by us [50].

### ELISA for IL-8

The secretion of IL-8 in the spent media of control and treated cells was measured by DuoSet ELISA kit for human IL-8 (R&D Systems, Minneapolis, MN), according to the manufacturer's instructions as described previously by us [24].

### Measurement of Bcl10 promoter activity

A 1310 bp fragment of the **5**′-untranslated region (p-Bcl1310) of Bcl10 gene cloned earlier [24] into the pGL2 reporter plasmid (Promega, Madison, WI) was transfected into Caco-2 cells were using Lipofectamine 2000 (Invitrogen). Twenty-four h after transfection, cells were treated with PAF for an additional 24 h period. Subsequently, promoter activity was determined by measuring luciferase activity and normalizing with the corresponding β-galactosidase activity, according to the procedure described previously [24].

### Ubiquitination of IKK-γ

Caco-2 cells were transfected with pcDNA3 expression vectors encoding Myc-tagged IKKγ and HA-tagged ubiquitin (kind gifts from PC Lucas of the University of Michigan Medical School, Ann Arbor, MI) and then treated with PAF and/or LA-CS as described above. After immunoprecipitating the IKKγ from the cell lysate with anti-Myc antibody (Santa Cruz, CA), the protein was assayed for ubiquitination by Western blotting with anti-HA antibody (Santa Cruz, CA).

### Statistical analyses

The data presented are mean ± SEM of 3–4 independent experiments. Difference between controls versus various treatments was analyzed using one-way ANOVA, with Dunnett’s multiple comparison tests for repeated comparisons to the control. Differences were considered significant at *P*<0.05.
