# Correction: *Lactobacillus acidophilus* Alleviates Platelet-Activating Factor-Induced Inflammatory Responses in Human Intestinal Epithelial Cells

**DOI:** 10.1371/journal.pone.0142593

**Published:** 2015-11-05

**Authors:** Alip Borthakur, Sumit Bhattacharyya, Anoop Kumar, Arivarasu Natarajan Anbazhagan, Joanne K. Tobacman, Pradeep K. Dudeja

The top panel of western blots in Fig 4C is mistakenly duplicated in Fig 5A. The authors have provided a corrected version of Fig 5A, which includes the correct upper panel. The raw blots for Figs 4C and 5A are available as Supporting Information. Additionally, there is an error in the caption for Fig 4. The immunoblotting was performed in NCM460 cells, not in Caco-2 cells. Please see the correct caption here.

**Fig 4:**
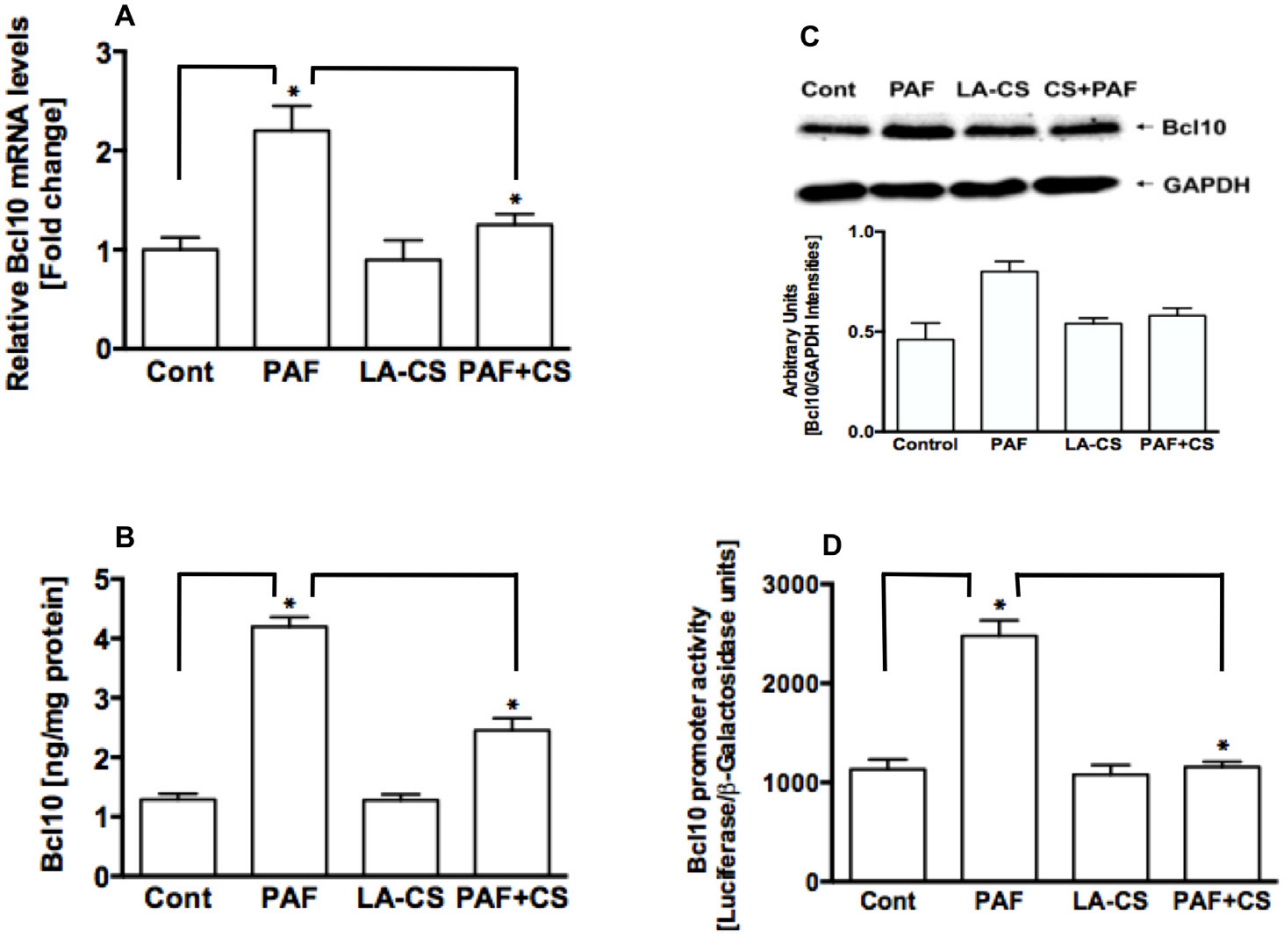
*L*. *acidophilus* culture supernatant does not alter Bcl10 expression but attenuates PAF-induced increase in Bcl10 expression in IECs. (**A**) relative levels of Bcl10 mRNA in control or PAF ± LA-CS-treated NCM460 cells measured by real-time RT-PCR as described in Methods (n = 3, *P<0.05); (**B**) Bcl10 protein levels measured by ELISA in control or PAF ± LA-CS-treated NCM460 cells (n = 3, *P<0.05); (**C**) representative blot (n = 3) of Bcl10 protein levels measured by immunoblotting (upper panel) in control or PAF ± LA-CS-treated NCM460 cells and densitometric analysis of the relative band intensities (lower panel); (**D**) Bcl10 promoter activity in control or PAF ± LA-CS-treated NCM460 cells (n = 4, *P<0.05).

**Fig 5:**
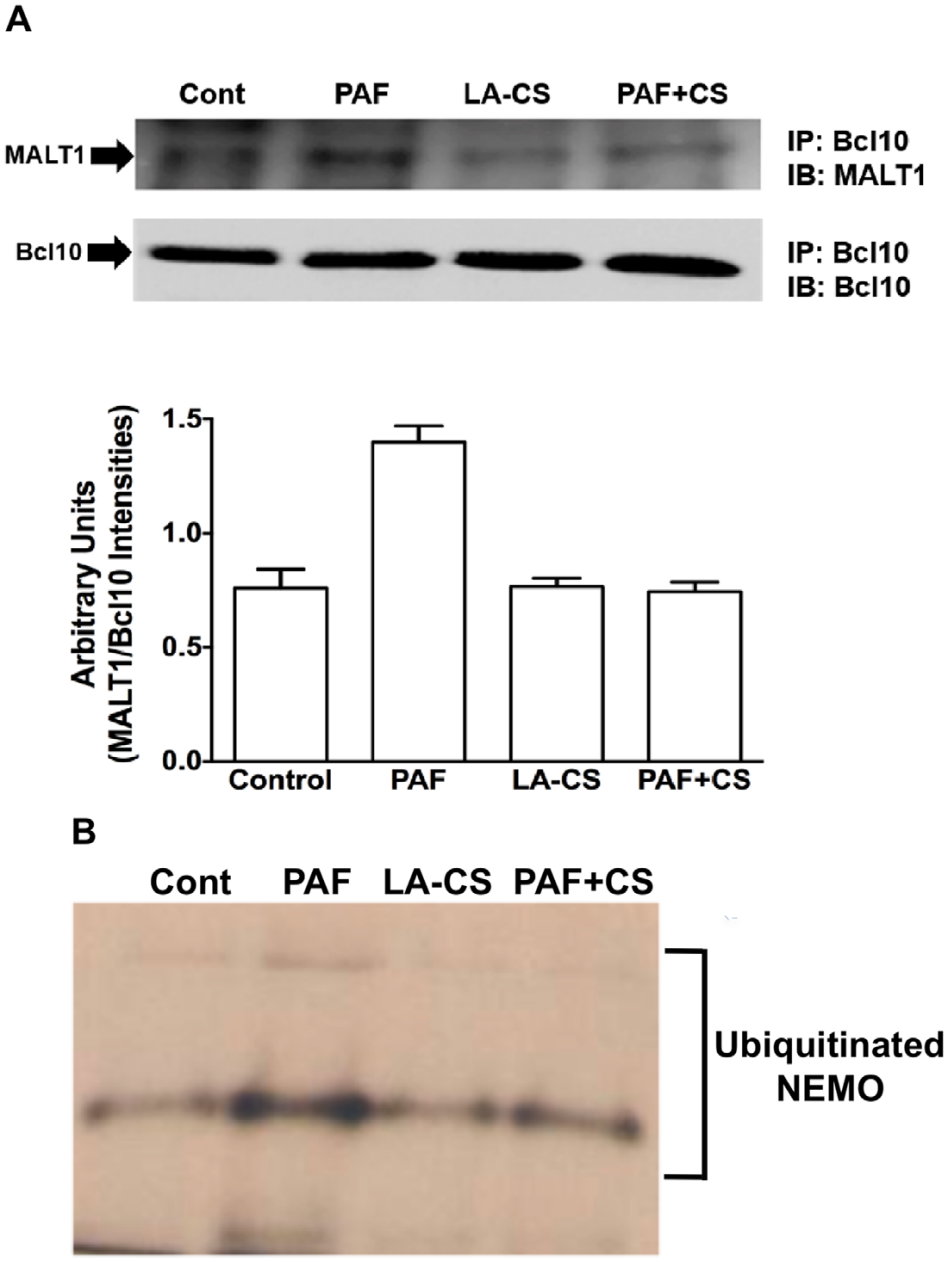
*L*. *acidophilus* culture supernatant attenuates PAF-induced Bcl10 interaction with MALT1 and ubiquitination of IKKγ (NEMO). (**A**) Cell lysates of control or PAF ± LA-CS-treated NCM460 cells, containing equal amounts of proteins, were used to immunoprecipitate (IP) MALT1 with anti-Bcl10 antibody. Immunoprecipitates were subjected to SDS-PAGE and probed with anti-MALT1 antibody in immunoblotting (IB). After stripping with 0.2N NaOH, blots were re-probed with anti-Bcl10 antibody; upper panel: representative blot of 3 independent experiments; lower panel: densitometric analysis of relative band intensities; (**B**) Caco-2 cells co-transfected with expression vectors encoding pcDNA3-IKKγ-Myc and pcDNA3-ubiquitin-HA were untreated or treated with PAF ± LA-CS as described in Methods. After purification by anti-Myc antibody immunoprecipitation, the IKKγ protein was assayed for ubiquitination by Western blotting with anti-HA antibody.

## Supporting Information

S1 Blots The raw blots for Figs 4C and 5A. (ZIP)
