# Supplementary figures and images for: Correction: Lactobacillus acidophilus Alleviates Platelet-Activating Factor-Induced Inflammatory Responses in Human Intestinal Epithelial Cells
Source: PLoS One. 2015 Nov 5;10(11):e0142593. doi: 10.1371/journal.pone.0142593 (PMC4635021; doi:10.1371/journal.pone.0142593)

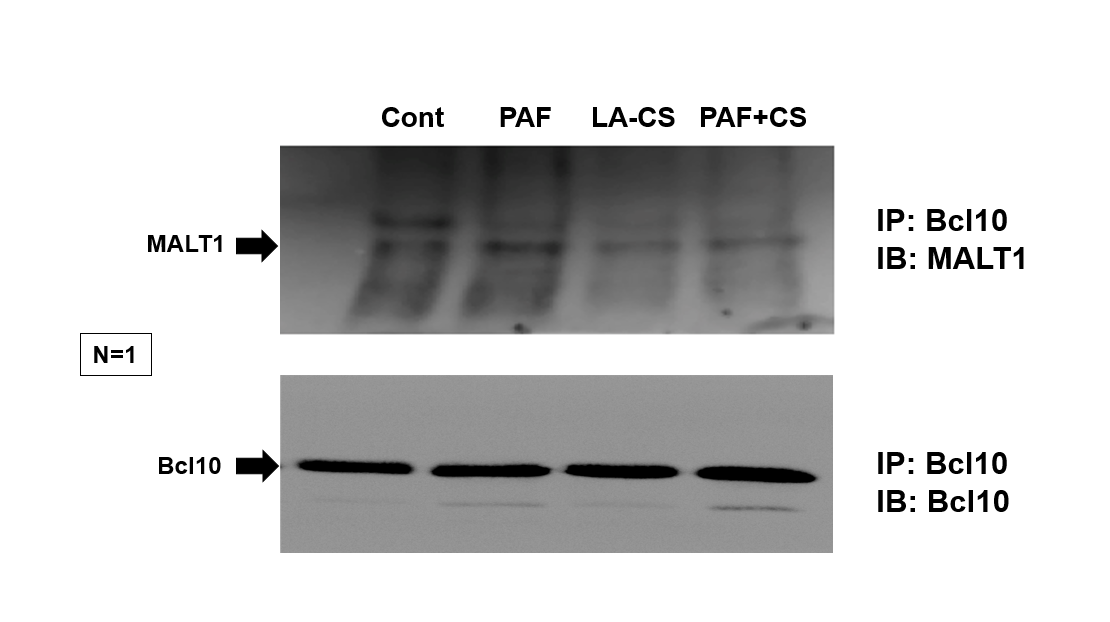

Supplement: S1 Blots — (ZIP) [file pone.0142593.s001.zip › Figure 5A Original Upper Lower Blots.tiff]

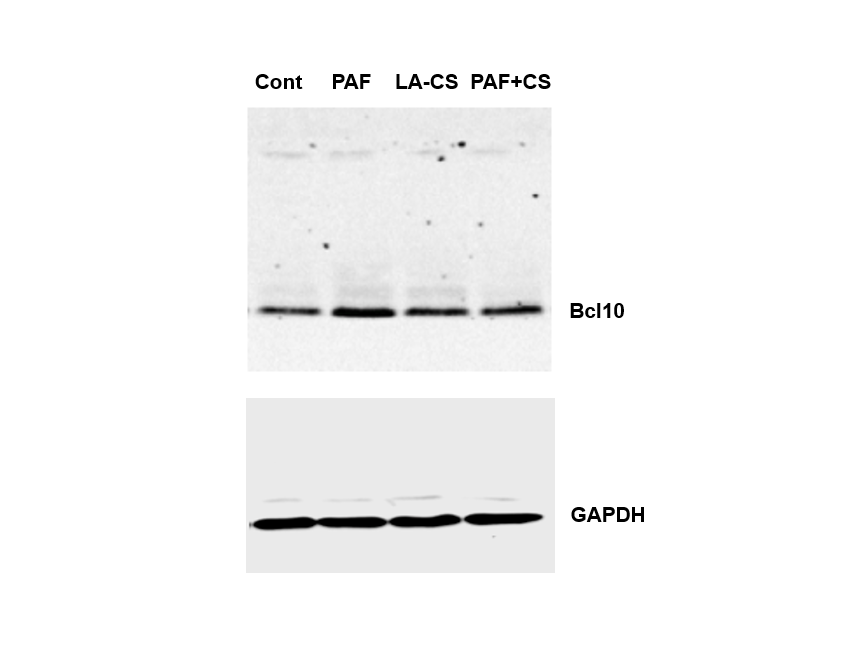

Supplement: S1 Blots — (ZIP) [file pone.0142593.s001.zip › Figure 4C Upper Lower Blots Original.tiff]
